# Evolving Spectrum of Dengue: A Two-Year Experience From a Tertiary Care Hospital in Pakistan

**DOI:** 10.7759/cureus.53817

**Published:** 2024-02-08

**Authors:** Fibhaa Syed, Mohammad Ali Arif, Valeed B Mansoor, Muhammad Usman, Saba Ali Arif

**Affiliations:** 1 Internal Medicine, Shaheed Zulfiqar Ali Bhutto Medical University, Islamabad, PAK; 2 Internal Medicine, Pakistan Institute of Medical Sciences, Islamabad, PAK; 3 Ophthalmology, Pakistan Institute of Medical Sciences, Islamabad, PAK

**Keywords:** complications of dengue fever, outcome of dengue, characteristics of dengue patient, expanded dengue syndrome, dengue virus infection

## Abstract

Objective: This study focused on examining the clinical manifestations, disease severity, and outcomes among cases of dengue fever (DF) confirmed through serological testing. The study specifically targeted individuals admitted to a tertiary care hospital in Islamabad, Pakistan.

Methodology: This prospective observational study at the Pakistan Institute of Medical Sciences, Islamabad, Pakistan, tracked 1,003 patients from admission to discharge or death between August 2022 and November 2023. Patients were monitored, and admission criteria were established based on the identification of warning signs. The data collection process encompassed gathering demographic information, documenting clinical symptoms, and utilizing a severity classification system for the disease. Outcome measures comprised the duration of critical illness, length of hospital stay, overall outcomes (discharge or mortality), and the assessment of complications. The collected data were analyzed using IBM Statistical Package for the Social Sciences (SPSS) software version 22.0 (IBM Corp., Armonk, NY).

Results: Baseline characteristics revealed a male predominance (67.8%), with an average age of 35.77 years, and common comorbidities such as hypertension (9.3%) and diabetes mellitus (7.3%). Dengue fever was most prevalent among patients whose blood group was B+ (15.0%). Nonstructural protein 1 (NS1) was positive in 73.4% of the cases. Fever was the predominant complaint in 98.0% of instances. Common bleeding manifestations included epistaxis, gum bleeding, and hematemesis. About 52.20% of cases were observed to have severe thrombocytopenia at admission. Hospital-related aspects demonstrated a mean stay of 3.35 days, a critical phase lasting 1.68 days, and rare complications like expanded dengue syndrome (2.2%). Encouragingly, 98.9% of patients were discharged, 0.4% were shifted, and 0.7% succumbed to the disease.

Conclusion: This study comprehensively analyzes the demographic and clinical aspects of DF, emphasizing a male predominance and the fact that fever was the most common presenting complaint. The duration of hospitalization revealed a brief mean stay, a short critical phase, and low complication rates, with a high discharge rate suggesting positive outcomes.

## Introduction

Dengue virus (DENV), a formidable viral threat disproportionately impacting the developing world, exists in more than 100 countries worldwide, with nearly half of the global population residing in endemic areas [1]. Annually, an estimated 390 million cases of DENV infection emerge, affecting about 2.5 billion people globally. The alarming reality is that the incidence of this pandemic-prone disease has surged by a factor of 30 in the last five decades [2]. This surge places a substantial health and economic burden on the WHO South-East Asia region, now marked by hyperendemicity for multiple DENV serotypes/genotypes (DENV-1 to DENV-4).

Belonging to the genus *Flavivirus *within the *Flaviviridae *family, DENV is characterized by its small, spherical, lipid-enveloped structure housing a single-stranded, positive-sense ribonucleic acid genome. Its transmission primarily occurs through anthropophilic mosquitoes of the *Aedes *(*Ae.*)* *genus, such as *Ae. aegypti* and *Ae. albopictus*, as well as primatophilic arboreal *Aedes spp.* like *Ae. niveus s.l.* in Asia and *Ae. furcifer* in Africa [3].

Each epidemic cycle changes the predominant serotype/genotype, often coinciding with severe disease and heightened transmission [4]. Clinically, dengue spans a spectrum from asymptomatic infection to severe manifestations, with an untreated mortality rate of 20% [2,5]. Lifelong immunity to the homotypic serotype follows a primary DENV infection, but heterotypic infections may lead to increased disease severity due to pre-existing immune memory from the primary infection [6].

The clinical manifestations of dengue are well documented, encompassing a range from benign dengue fever (DF) to severe forms affecting multiple organs, including the liver, muscles, kidneys, heart, and nervous system. Uncommon clinical presentations, such as encephalopathy, encephalitis, fulminant hepatitis, splenomegaly, and ocular complications, have also been reported [6].

This article delves into the presentation, clinical course, disease severity, and outcomes of dengue patients, focusing on evolving clinical parameters and providing a comprehensive understanding of this viral menace in a tertiary care hospital setting.

## Materials and methods

Study design, setting, and patients

This prospective observational study was conducted at the Pakistan Institute of Medical Sciences in Islamabad, Pakistan, a distinguished teaching hospital affiliated with the Shaheed Zulfiqar Ali Bhutto Medical University in Islamabad, Pakistan. A total of 1,003 patients were tracked from admission to discharge or death from August 2022 until November 2023. Using data from the WHO [7] reporting 25,932 cases in Pakistan from January to September 2022, a margin of error of 5% and a confidence interval of 95%, after incorporating these in Raosoft online sample size calculator (Raosoft Inc., Seattle, WA), a sample size of 379 patients was calculated. The admission criteria involved identifying warning signs and severe cases. Patients presenting warning signs, in shock, with comorbidities, and those exhibiting expanded dengue syndrome were admitted. The follow-up process was conducted meticulously, adhering to ethical research practices. Individuals unable to communicate or unwilling to provide consent were excluded.

Clinical and laboratory investigations

The diagnosis of DF was established through a comprehensive approach incorporating clinical and laboratory parameters. A patient was considered to have DF if there was a reported history of fever accompanied by at least two of the following symptoms: anorexia/nausea/rashes/aches and pains, the presence of warning signs, leucopenia, and a positive tourniquet test. Additionally, confirmation of DF required laboratory evidence, achieved by detecting the nonstructural protein 1 (NS1) antigen or IgM serology of dengue using an enzyme-linked immunosorbent assay (ELISA). Blood samples collected in aseptic ethylenediaminetetraacetic acid (EDTA) tubes were subjected to the analysis of anti-dengue antibodies using IgM capture ELISA and IgG ELISA [2,4].

The patients underwent a complete blood count analysis at baseline and after every 12 or 24 hours as required by their clinical status, specifically observing the platelet count and the hematocrit (HCT) percentage. Their liver and renal function tests were also performed. Bleeding manifestations, the platelet count at the bleeding episode, and the bleeding site were monitored.

Warning signs included abdominal pain or tenderness, persistent vomiting, clinical fluid accumulation, mucosal bleeding, lethargy and restlessness, liver enlargement >2 cm, and a laboratory report showing an increase in HCT concurrent with a rapid decrease in platelet count [3]. Patients with warning signs, shock, comorbidities, and expanded dengue syndrome were admitted.

Data collection

The data for this study were collected using a structured questionnaire. Data were recorded and managed using Microsoft Excel spreadsheets (Microsoft Corp., Redmond, WA) to facilitate organized and efficient data collection and analysis. Demographic details, encompassing age, gender, comorbidities, BMI, blood group, and dengue history, were gathered. The severity of thrombocytopenia was also assessed and classified into mild, moderate, severe, and very severe categories based on specific platelet count thresholds. Mild thrombocytopenia ranges from 100,000 to 150,000 cells/mm³, moderate from 50,000 to 100,000 cells/mm³, severe from 20,000 to 50,000 cells/mm³, and very severe below 20,000 cells/mm³. Concurrently, clinical data documenting presenting symptoms allowed for the categorization of symptomatic DENV infections into DF and dengue hemorrhagic fever (DHF). Further classification of DHF into four severity grades, defining grades 3 and 4 as dengue shock syndrome (DSS), was based on a comprehensive grading system outlined in Table 1.

**Table 1:** Grading of dengue fever and associated laboratory parameters DF: dengue fever; DHF: dengue hemorrhagic fever; DSS: dengue shock syndrome

DF/DHF/DSS	Grade	Symptoms	Laboratory
DF	-	Fever with two or more signs: headache, retro-orbital pain, arthralgias, and myalgias	Leucopenia and occasional thrombocytopenia may be present. No plasma loss.
DHF	1	The abovementioned signs plus a positive tourniquet test	Thrombocytopenia ≤100,000, hematocrit rise ≥20 %
	2	The abovementioned signs plus spontaneous bleeding	Thrombocytopenia ≤100,000, hematocrit rise ≥20 %
DSS	3	The abovementioned signs plus circulatory failure, weak pulse, hypotension, and restlessness	Thrombocytopenia ≤100,000, hematocrit rise ≥20 %
	4	Profound shock with undetectable blood pressure and pulse	Thrombocytopenia ≤100,000, hematocrit rise ≥20 %

Additionally, the total duration of fever and the specific day of illness on which the patient was admitted were meticulously recorded.

Outcome measures

Outcome measures were comprehensive, encompassing the duration of critical illness, hospital stay, disease severity, overall outcomes (discharge or death), and a thorough evaluation of complications such as rhabdomyolysis, encephalitis, hepatitis, and acute kidney injury.

Statistical analysis

Data collected on questionnaires were coded and entered into IBM Statistical Package for the Social Sciences (SPSS) software version 22.0 (IBM Corp., Armonk, NY). Categorical variables were expressed as frequencies and percentages, whereas continuous variables were expressed as the mean, standard deviation (SD), interquartile range (IQR), and median.

Ethical considerations

Hospitalized patients with DF were enrolled after approval from the ethical review board of Shaheed Zulfiqar Ali Bhutto Medical University, Islamabad, Pakistan (approval number: F.1-1/2015-ERB/SZABMU/1031) granted on July 7, 2022.

## Results

Table 2 summarizes the baseline characteristics of 1,003 study participants, with 32.2% females and 67.8% males.

**Table 2:** Baseline characteristics of the study population (N=1,003) N: number

Variables			N (%)
Gender		Female	323 (32.2)
		Male	680 (67.8)
Age (in years (Mean±SD))			35.77±15.26
	<30 years		452 (45.1)
	31 to 50 years		380 (37.9)
	51 to 70 years		153 (15.3)
	>70 years		18 (1.8)
BMI (kg/m^2 ^(Mean±SD))			25.83±3.43
Comorbidities		Diabetes mellitus	73 (7.3)
		Hypertension	93 (9.3)
		Ischemic heart disease	27 (2.7)
		Others	46 (4.6)
Blood group		A+	104 (10.4)
		A-	10 (1.0)
		B+	150 (15.0)
		B-	12 (1.2)
		AB+	34 (3.4)
		AB-	8 (0.8)
		O+	127 (12.7)
		O-	39 (3.9)
		Not reported	519 (51.7)

The average age of participants was 35.77 years (SD=15.26), and the mean BMI was 25.83 kg/m² (SD=3.43). Comorbidities included hypertension (9.3%), diabetes mellitus (7.3%), and ischemic heart disease (2.7%). Blood group distribution varied, with patients with B+ blood group being the affected (15.0%).

Table 3 outlines the dengue serology distribution, with 73.4% of the participants testing positive for NS1 only.

**Table 3:** Clinical presentation and laboratory parameters of the study group N: number; IQR: interquartile range

Variables		N (%)
Dengue diagnostics	IgG only	40 (4.0)
	IgM only	71 (7.1)
	Nonstructural protein 1 (NS1) only	736 (73.4)
	IgG + IgM	52 (5.2)
	IgG + NS1	6 (0.6)
	IgM + NS1	62 (6.2)
	IgG + IgM + NS1	26 (2.6)
	Negative	10 (1.0)
Presenting complaints	Fever	983 (98.0)
	Nausea/vomiting	709 (70.7)
	Abdominal pain	294 (29.3)
	Body ache	602 (60.0)
	Headache	244 (24.3)
	Cough	67 (6.7)
	Retro-orbital pain	62 (6.2)
Febrile period at admission (days (Mean±SD))		4.70±2.76
Number of days at which fever is resolved (Mean±SD)		3.45±2.07
Laboratory parameters (Median (IQR))	Platelets (per μL (N=1,003))	15,000.0 (12,000.0-18,000.0)
	Creatine phosphokinase (CPK) (U/L (N=3))	870.0 (395.0-1345.0)
	Alanine transaminase (ALT) (U/L (N=109))	220.0 (98.0-342.0)
	C-reactive protein (CRP) (mg/dL (N=1,003))	220.5 (76.0-365.0)
	Total leukocyte count (TLC) (cells per μL (N=1,003))	1,809.5 (1,200.0-2,419.0)
	Hemoglobin (Hb) (g/dL (N=1,003))	8.7 (7.5-10.0)
	Hematocrit (HCT) (% (N=1,001))	24.5 (20.0-29.0)
	Neutrophils (% (N=987))	73.0 (56.0-90.0)
	Lymphocytes (% (N=975))	6.0 (2.0-10.0)
	Monocytes (% (N=973))	4.2 (1.4-7.0)

The predominant presenting complaint was fever (98.0%), followed by nausea/vomiting (70.7%). The mean febrile period at admission was 4.70±2.76 days.

**Figure 1:**
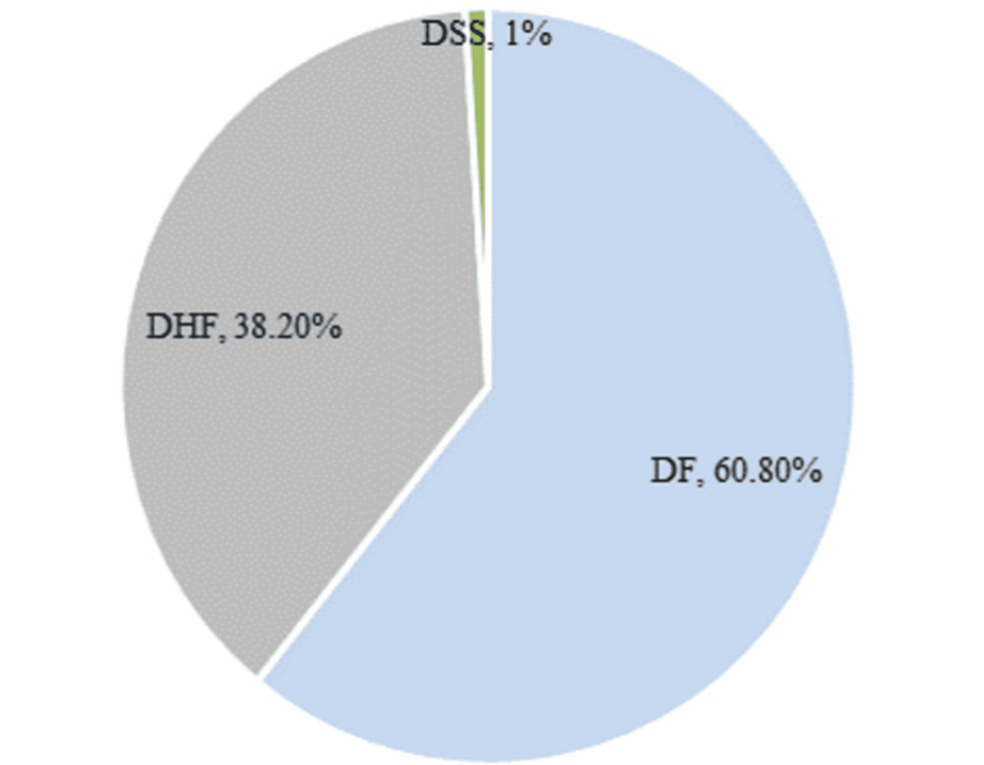
The severity of dengue fever among the study participants DSS: dengue shock syndrome; DHF: dengue hemorrhagic fever; DF: dengue fever

Based on the severity grading, 11.70% and 26.50% of the cases demonstrated DF-1 and DF-2, respectively. The more severe classifications, DF-3 and DF-4, were less common, comprising 0.90% and 0.10%, respectively. Grades 1 and 2 were classified as DHF (n=383), and grades 3 and 4 were categorized as DSS (n=10). This is illustrated in Figure 1.

**Figure 2:**
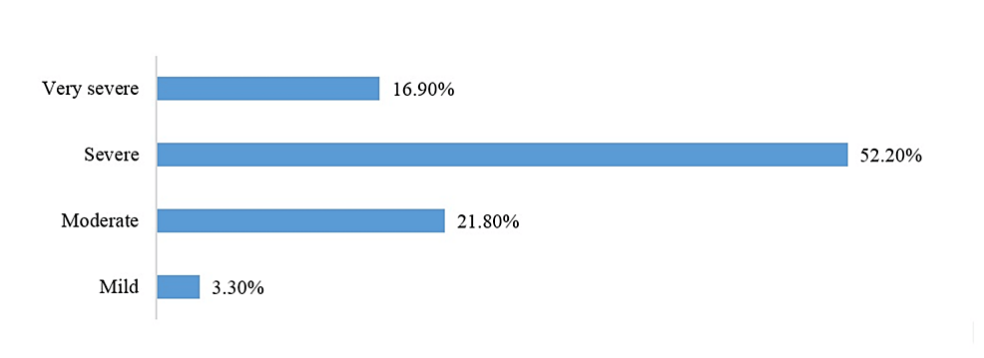
Severity of thrombocytopenia in the enrolled patients

As illustrated in Figure 2, thrombocytopenia at admission in the study cohort revealed a predominant severity, with 52.20% of the study participants categorized as severe cases and 16.90% categorized as very severe.

Moderate thrombocytopenia was observed in 21.80%, mild in 3.30%, and a smaller proportion, 5.70%, presented with normal platelet counts.

Table 4 details bleeding manifestations among the study participants at admission and during the hospital stay.

**Table 4:** Bleeding manifestations among the study participants

Occurrence of bleeding	Site of bleeding	Frequency (%)
At admission	Bleeding per rectum (PR)	23 (2.3)
	Hemoptysis	18 (1.8)
	Gums	68 (6.8)
	Hematuria	35 (3.5)
	Gastrointestinal (GI) bleed	4 (0.4)
	Epistaxis	84 (8.4)
	Bleeding per vaginum (PV)	35 (3.5)
	Melena	10 (1.0)
	Hematemesis	41 (4.1)
During hospital stay	PV	8 (0.8)
	PR bleed	12 (1.2)
	GI bleed	4 (0.4)
	Gums	20 (2.0)
	Hematemesis	14 (1.4)
	Melena	12 (1.2)
	Hemoptysis	4 (0.4)
	Hematuria	13 (1.3)
	Epistaxis	20 (2.0)

At admission, common bleeding manifestations included epistaxis (8.4%), gum bleeding (6.8%), and hematemesis (4.1%). During hospitalization, occurrences of bleeding included epistaxis (2.0%), gum bleeding (2.0%), and hematuria (1.3%).

Table 5 illustrates the hospital-related aspects of the study.

**Table 5:** Hospital stay duration, nature of complications, and outcomes of the study N: number

Variables		N (%)
Duration of hospital stay in days (N=1,003) (Mean±SD)		3.35±2.03
Duration of critical phase (N=387) (Mean±SD)		1.68±1.00
Complications	Acute kidney injury (AKI)	1 (0.09)
	AKI/diabetic ketoacidosis (DKA)	1 (0.09)
	Dengue encephalitis	3 (0.29)
	Dengue hepatitis	6 (0.59)
	Dengue hepatitis and rhabdomyolysis	2 (0.19)
	Dengue shock	9 (0.89)
Expanded dengue syndrome	Yes	22 (2.2)
	No	981 (97.8)
Outcomes	Discharged	992 (98.9)
	Shifted	4 (0.4)
	Death	7 (0.7)

The mean duration of hospital stay was 3.35±1.68 days for those affected. Complications were rare, with 97.8% having no complications. Encouragingly, 98.9% of patients were discharged, 0.4% were shifted, and 0.7% succumbed to the disease.

## Discussion

Dengue virus infection is a global health threat, particularly with the rise of severe conditions like DHF and DSS, which substantially strain healthcare resources [8]. This concern is especially pertinent in Pakistan, where dengue has reached epidemic proportions, necessitating urgent policy interventions to control the associated mortality and morbidity [9]. In the specific context of a tertiary care hospital in Islamabad, during the dengue epidemic of 2022, a significant portion of the admitted patients (45.1%) were 30 years of age or younger. The mean age of these dengue patients was 35.77±15.26 years. Comparatively, a retrospective study conducted in Rawalpindi during the 2019 epidemic, involving 12,192 dengue cases, highlighted that a major segment (30.4%) belonged to the 31-40 age group, with a median age of 36±14.6 years [10].

Following the WHO 1997 classification, DF was confirmed in 610 patients (60.80%), with 383 patients (38.2%) diagnosed with DHF and only 10 cases (0.9%) confirmed as DSS at the Pakistan Institute of Medical Sciences which is a tertiary care hospital. Parallel to our findings, a Malaysian study adopting the WHO 2009 classification reported a higher frequency of diagnosed DF cases at 88.1%, with only 11.1% and 0.8% confirmed as DHF and DSS, respectively [11]. Similarly, a study by Shahid et al. documented the confirmation of DF in 54.8% of patients, and diagnosis of DHF in 43%, with a mere 2.2% of confirmed cases of DSS in the tertiary care hospitals of Rawalpindi Medical University (RMU) [10]. The higher incidence of DHF and DSS in local studies, including our research and Shahid et al.'s, in comparison to the Malaysian study, may be attributed to factors such as poor adherence to hygienic practices, a lack of awareness, and delays in seeking healthcare services for unusual symptoms.

The mean duration of hospital stay in our study was 3.35±2.03 days, with the critical phase lasting 1.68±1.00 days for affected individuals. In contrast, a local study reported a shorter duration, specifically 1.28±0.67 days [10]. Interestingly, the Malaysian study, aligning with our findings, also reported a prolonged mean length of hospital stay of 4.88±2.74 days [11]. This variation could be influenced by differences in healthcare practices, patient management protocols, or the severity of cases encountered in these settings.

Regarding dengue diagnostic distribution, a significant proportion of patients in our study, specifically 73.4%, tested positive for NS1 only. The primary presenting complaint was fever, prevalent in 98.0% of cases, with an average febrile period of 4.70±2.76 days at admission. Other common symptoms included nausea/vomiting (70.7%), abdominal pain (29.3%), body ache (60.0%), headache (24.3%), cough (6.7%), and retro-orbital pain (6.2%). Additionally, notable bleeding manifestations primarily encompass epistaxis, gum bleeding, and hematemesis. Comparatively, a study conducted in Dhaka by Islam et al. [12] reported fever as the predominant symptom (94.56%), followed by myalgia, rashes (58.58%), bone pain (54.39%), headache (48.95%), petechiae (42.89%), vomiting (32.01%), abdominal pain (29.71%), and cough (24.90%). Gastrointestinal tract bleeding (13.18%), diarrhea (11.92%), gum bleeding (10.25%), hematuria (09.41%), and retro-orbital pain (08.58%) were also observed. These findings underscore the diverse clinical presentation of DF, with variations possibly influenced by regional or population-specific factors.

Thrombocytopenia at admission within our study cohort displayed a substantial severity spectrum, with 52.20% of the patients categorized as severe cases and an additional 16.90% categorized as very severe cases. Moderate thrombocytopenia was observed in 21.80%, while mild cases accounted for 3.30%, and a smaller proportion, 5.70%, presented with normal platelet counts. These findings align with a study reporting a similar frequency of thrombocytopenia (57.95%) in 478 dengue cases [12]. In contrast, an Indian study focusing on 300 children with DF reported a higher prevalence of thrombocytopenia (92%). Within this thrombocytopenic group, 32% exhibited mild thrombocytopenia, 55.7% had moderate thrombocytopenia, and 11.5% presented with severe thrombocytopenia [13].

Understanding the significance of blood groups in DF cases is crucial, as various studies have explored potential correlations between specific blood types and susceptibility to severe forms of dengue. Existing research suggests that individuals with blood type O may exhibit increased susceptibility to severe manifestations of dengue, while blood type A could be associated with a lower risk [14]. In the present study, the distribution of blood groups among confirmed dengue cases revealed diverse patterns, with 51.7% of patients not reporting their blood group. Among the reported cases, A+, B+, O+, AB+, A-, B-, AB-, and O- constituted 10.4%, 15.0%, 12.7%, 3.4%, 1.0%, 1.2%, 0.8%, and 3.9%, respectively. All individuals included in this blood group analysis were confirmed dengue cases. Examining the distribution of blood groups contributes to exploring potential correlations between blood types and the susceptibility or severity of dengue infections. Previous studies associated blood group AB with a higher risk of developing DHF, while blood group O was linked to a lower risk [15]. However, the current study found that patients with blood group O were significantly less affected by clinically apparent DENV infections, emphasizing the nuanced nature of these associations and the need for continued exploration in diverse populations. Integrating blood group information into future studies has the potential to offer valuable insights into host factors influencing the severity of dengue cases.

Complications were infrequent, with 97.8% of patients experiencing none, and the notable complications included dengue shock and hepatitis. Only 2.2% of patients reported having expanded dengue syndrome. Encouragingly, most (98.9%) patients were discharged, while a minimal proportion (0.4%) were shifted, and 0.7% succumbed to the disease. A Taiwanese study reported a comparable mortality rate [16]. These outcomes highlight the overall positive prognosis for most dengue patients in our study, emphasizing the importance of timely medical intervention and management strategies in mitigating severe complications and improving survival rates.

The management of DF primarily involves supportive care, as there is no specific antiviral treatment for the infection. Patients are advised to stay hydrated to counteract fluid loss due to fever and potential bleeding manifestations. Pain relievers such as acetaminophen are recommended for managing fever and body aches while avoiding non-steroidal anti-inflammatory drugs (NSAIDs) like ibuprofen, as they may contribute to bleeding risks [17]. In severe cases, hospitalization may be necessary for close monitoring and intravenous fluid administration to maintain adequate fluid balance. Patients with signs of DHF or DSS, characterized by severe bleeding, organ damage, and shock, require immediate medical attention. Blood transfusions and other supportive measures may be implemented based on the severity of the condition. Timely medical intervention is crucial in preventing complications and ensuring a favorable outcome for dengue patients.

The study contributes significantly to the understanding of dengue fever by thoroughly examining its demographic and clinical aspects, providing valuable insights into symptoms, serology distribution, thrombocytopenia, complications, and outcomes. Despite its single-center focus, which might limit the generalizability of findings to milder cases not requiring hospitalization, the study's emphasis on in-depth exploration offers crucial perspectives into the experiences of hospitalized patients during the 2022 dengue epidemic in Islamabad. This detailed exploration establishes a strong foundation for future research and interventions in dengue management. However, the single-center approach introduces a potential selection bias, emphasizing the need for caution in generalizing the findings to the broader population experiencing milder forms of dengue.

## Conclusions

The study provides a comprehensive insight into the demographic and clinical characteristics of dengue fever, highlighting a notable predominance among males. The most common symptoms, particularly fever, offer a detailed understanding of the disease presentation. The hospitalization experience indicates a mean stay of 3.35 days, with a 1.68-day critical phase and rare complication rate (2.2%). A high discharge rate reflects a generally positive prognosis. 

## References

[1] Biswal S, Reynales H, Saez-Llorens X (2019). Efficacy of a tetravalent dengue vaccine in healthy children and adolescents. N Engl J Med.

[2] (2023). Dengue and severe dengue. December.

[3] Achee NL, Gould F, Perkins TA (2015). A critical assessment of vector control for dengue prevention. PLoS Negl Trop Dis.

[4] Tsheten T, Gray DJ, Clements AC, Wangdi K (2021). Epidemiology and challenges of dengue surveillance in the WHO South-East Asia Region. Trans R Soc Trop Med Hyg.

[5] Kouser S, Abbas FF, Naeem Effendi F (2020). Assessment of the association of ABO blood group in dengue fever diagnosed patient in tertiary care hospital. Int J Endorsing Health Sci Res.

[6] Estofolete CF, de Oliveira Mota MT, Bernardes Terzian AC (2019). Unusual clinical manifestations of dengue disease - real or imagined?. Acta Trop.

[7] World Health Organization (13 October 2022 (2023). Dengue - Pakistan. https://www.who.int/emergencies/disease-outbreak-news/item/2022-DON414.

[8] Khalil MA, Tan J, Khalil MA, Awan S, Rangasami M (2014). Predictors of hospital stay and mortality in dengue virus infection-experience from Aga Khan University Hospital Pakistan. BMC Res Notes.

[9] Ahsan T (2008). Dengue fever: a regular epidemic?. J Pak Med Assoc.

[10] Shahid R, Umar M, Zafar RB, Zeb S, Ambreen S, Akram MO (2020). Comorbidity of COVID-19 related fatalities in tertiary care hospitals of Rawalpindi, Pakistan. J Rawalpindi Med Coll.

[11] Mallhi TH, Khan AH, Sarriff A, Adnan AS, Khan YH (2017). Determinants of mortality and prolonged hospital stay among dengue patients attending tertiary care hospital: a cross-sectional retrospective analysis. BMJ Open.

[12] Islam S, Hasan MN, Kalam SB, Islam MS, Hasan MJ, Sami CA, Chowdhury FR (2022). Clinical profile, severity spectrum, and hospital outcome of dengue patients in a tertiary care hospital in Dhaka city. Cureus.

[13] Selvan T, Joy LP, Souza D, Swamy N, Kumar M (2015). Prevalence and severity of thrombocytopenia in dengue fever in children. Sch J App Med Sci.

[14] Lien CE, Chou YJ, Shen YJ, Tsai T, Huang N (2021). A population-based cohort study on chronic comorbidity risk factors for adverse dengue outcomes. Am J Trop Med Hyg.

[15] Ravichandran S, Ramya SR, Kanungo R (2019). Association of ABO blood groups with dengue fever and its complications in a tertiary care hospital. J Lab Physicians.

[16] Lee YH, Hsieh YC, Chen CJ, Lin TY, Huang YC (2021). Retrospective seroepidemiology study of dengue virus infection in Taiwan. BMC Infect Dis.

[17] Islam A, Cockcroft C, Elshazly S (2022). Coagulopathy of dengue and COVID-19: clinical considerations. Trop Med Infect Dis.

